# Supplementary material for: Identifying patients with multidrug-resistant tuberculosis who may benefit from shorter durations of treatment
Source: PLoS One. 2023 Oct 5;18(10):e0292106. doi: 10.1371/journal.pone.0292106 (PMC10553332; doi:10.1371/journal.pone.0292106)
Supplement: S1 File — (DOCX) [file pone.0292106.s001.docx]

**Supplementary materials for:**

**Identifying patients with multidrug-resistant tuberculosis who may benefit from shorter durations of treatment**

Table of Contents

[Supplement 1. Eligibility criteria, search strategy, and quality assessment. 2](#_Toc132363607)

[Supplement 2. Completeness of information, quality of all studies in the IPD, and their inclusion status in this analysis. 4](#_Toc132363608)

[Supplement 3. Detail on models for ecological level analysis. 5](#_Toc132363609)

[Supplement 4. Detail on descriptions and definitions of baseline patient characteristics, drug susceptibility testing, and treatments used. 5](#_Toc132363610)

[Supplement 5. Detail on multivariate imputation by chained equations (MICE). 6](#_Toc132363611)

[Supplement 6. Detail on inverse probability of selection weights. 6](#_Toc132363612)

[Supplement 7. Detailed description of E-Values. 6](#_Toc132363613)

[Supplement 8. Detail on variable selection due to correlation in the multivariable model for the primary analysis. 7](#_Toc132363614)

[Supplemental Table S1. Comparison of patients in the IPD who were included and excluded from this analysis. 8](#_Toc132363615)

[Supplemental Table S2. Mean total treatment duration and total number of patients at each site included in the study population. 11](#_Toc132363616)

[Supplemental Table S3. Associations between deviation in treatment duration from site mean and patient characteristics, resistance categories, and drugs used from the full multivariable model with inverse probability of selection weights and without (primary analysis), including all variables listed. 14](#_Toc132363617)

[Supplemental Table S4. E-values for selected characteristics with largest effect estimates for deviation in treatment duration. 15](#_Toc132363618)

[Supplemental Table S5. Associations between deviation in treatment duration from site mean and patient characteristics, resistance categories, and drugs used by different combinations of subgroups, and their regression estimates and 95% confidence intervals (CI) from a multivariable linear mixed model including all variables listed (unless specified otherwise). 16](#_Toc132363619)

[Supplemental Figure S1. Forest plot of associations between deviation in treatment duration (in months) from site mean and patient characteristics, resistance categories, and drugs used analyzed using imputed outcomes for subjects with missing or planned duration. Estimates and 95% confidence intervals (CI) from regression using a multivariable linear mixed model, including all variables shown (n = 7885). 17](#_Toc132363620)

[References 18](#_Toc132363621)

# Supplement 1. Eligibility criteria, search strategy, and quality assessment.

**Eligibility criteria:** Included studies were those reporting original results with end of treatment outcomes (ie, success, failure or relapse, and death) for 25 or more adults (to avoid small series reporting unusual cases) with bacteriologically confirmed pulmonary multidrug resistant tuberculosis. Studies exclusively in children or of patients treated with short regimens were excluded as these were the topics of two concurrent individual patient data meta-analyses at time of original publication (*Lancet* 2018; **392**(10150): 821-34.)

**Search Strategy:**

Medline search (through Ovid)
MDR or XDR
1. exp multidrug resistant tuberculosis/ or exp extensively drug resistant tuberculosis/
2. (multidrug resistant tuberculosis or extensive* drug resistant tuberculosis or MDR-TB or XDR-TB).ti,ab,kw.
3. (tuberc* and (MDR or XDR or drug resistan* or multidrug resistan* or multi drug resistan* or poly drug resistan* or extensive*
drug resistan*)).ti,ab,kw.
Drugs
4. exp Fluoroquinolones/ or exp Quinolones/ or exp Levofloxacin/ or (fluoroquinolone* or quinolone* or levofloxacin or Levaquin
or moxifloxacin or Avelox).ti,ab,kw.
5. exp Kanamycin/ or exp Amikacin/ or exp Capreomycin/ or exp Aminoglycosides/ or (Kanamycin or Amikacin or Capreomycin or
(tuberc* and injectable*)).ti,ab,kw.
6. exp Pyrazinamide/ or exp Ethambutol/ or exp Cycloserine/ or exp Ethionamide/ or exp Prothionamide/ or (Pyrazinamide or
Ethambutol or para-aminosalicylic acid or Cycloserine or Ethionamide or Prothionamide).ti,ab,kw.
7. high dose.ti,ab,kw. and ((INH or isoniazid).ti,ab,kw. or exp isoniazid/)
Efficacy
8. exp Treatment Outcome/ or exp Prognosis/ or exp Death/ or exp Mortality/ or exp Treatment Failure/ or exp Survival/ or exp
Recurrence/ or exp Patient Dropouts/ or exp Patient Compliance/
9. (Treatment Outcome* or Prognosis or Death or Mortality or Treatment Failure or drug treatment failure or failure or Survival or
Recurrence or relapse or Patient Dropout* or dropout or non-compliance or compliance or efficacy or effective* or cure or
success* or default or adheren* or conversion* or microbiologic conversion or smear conversion or culture conversion or sputum
conversion).ti,ab,kw.
Toxicity
10. exp Treatment Outcome/ or exp Prognosis/ or exp Death/ or exp Mortality/ or exp Treatment Failure/ or exp Survival/ or exp
Recurrence/ or exp Toxicity Tests/ or exp Drug Tolerance/ or exp "Drug-Related Side Effects and Adverse Reactions"/
11. (Treatment outcome* or Prognosis or Death or Mortality or Treatment Failure or drug treatment failure or failure or Survival
or Recurrence or relapse or Toxicity Test* or toxicity or Drug Tolerance or toler* or intolerance or Side Effect* or Adverse Drug
Reaction* or adverse drug event* or adverse event* or adverse reaction* or safe* or drug safety).ti,ab,kw.
New drugs
12. (Bedaquiline or TMC-207 or delamanid or OPC-67683).ti,ab,kw.
Final steps
13. 1 or 2 or 3
14. 4 or 5 or 6 or 7
15. 8 or 9
16. 10 or 11

17. 13 and 14 and 15
18. 13 and 14 and 16
19. 12 and 13 and 15
20. 12 and 13 and 16
21. limit 17 to (humans and yr="2009 -Current")
22. limit 18 to (humans and yr="2009 -Current")
23. limit 19 to (humans and yr="2012 -Current")
24. limit 20 to (humans and yr="2012 -Current")
25. 21 or 22
26. 23 or 24
27. 25 or 26

(EmBase and the Cochrane Library were searched using the same strategy)

**Quality assessment:**

This is discussed in detail in Lancet 2018; 392: 821–34.

A checklist of seven indicators was developed (adapted from the Risk of Bias in Non-randomised Studies of Interventions (ROBINS-I) tool) to assess the quality of included studies. Two of these indicators were considered essential: 1) population selection using a census (all) or random selection approach; and 2) availability of drug susceptibility tests results to at least one fluoroquinolone and one second-line injectable (defined as any of amikacin, kanamycin, or capreomycin). For the remaining indicators, quality was judged to be adequate if the participation rate >80%, loss to follow-up <20%, treatment outcomes were defined according to published guidelines (Laserson and WHO 2013), and >90% of patient records had information about HIV infection, previous tuberculosis treatment, and age (as these are all important determinants of outcomes). Participation rates were based on the reported total number of eligible patients and the number enrolled, and we considered participation to be 100% if the investigators stated that all patients with multidrug-resistant tuberculosis were enrolled. Studies of high quality met both essential criteria and at least four of the other six. Studies of moderate quality met one of the two essential parameters and at least five in total. Remaining studies were considered of low quality.

| **No** | **Contact person (ref)** | **Sampling method** | **Info on SLI sensitivity** | **Info on FQ sensitivity** | **Participation rate** | **Lost to follow-up rate** | **Outcome definition** | **Info on age** | **Info on HIV** | **Info on TB treatment history** | **Quality** | **Included in this analysis** |
| --- | --- | --- | --- | --- | --- | --- | --- | --- | --- | --- | --- | --- |
| 1 | Ahmad^1^ | Census | 100.00% | 100.00% | 96.80% | 1.70% | Laserson | 100.00% | 100.00% | 100.00% | High | No |
| 2 | Ahuja^2^ | Random | 92.40% | 92.40% | 100.00% | 19.00% | Laserson | 100.00% | 80.00% | 100.00% | High | Yes |
| 3 | Anderson^3^ | Census | 100.00% | 100.00% | 100.00% | 12.40% | Neither | 100.00% | 100.00% | 90.50% | High | Yes |
| 4 | Bang^4^ | Census | 96.60% | 93.10% | 96.70% | 17.20% | Laserson | 100.00% | 100.00% | 100.00% | High | Yes |
| 5 | Barkane^5^ | Census | 100.00% | 100.00% | 100.00% | 15.60% | Laserson | 100.00% | 100.00% | 100.00% | High | Yes |
| 6 | Barry (Korea)^6,7^ | RCT | 100.00% | 100.00% | 92.70% | 10.50% | Laserson | 100.00% | 100.00% | 100.00% | High | Yes |
| 7 | Barry/Flood (Calif)^8^ | Unclear | 98.40% | 95.20% | 100.00% | 4.80% | WHO2013 | 98.40% | 100.00% | 100.00% | Moderate | Yes |
| 8 | Bonnet^9^ | Census | 93.30% | 93.30% | 100.00% | 41.30% | Laserson | 100.00% | 11.50% | 98.60% | High | Yes |
| 9 | Brode^10^ | Census | 100.00% | 100.00% | 100.00% | 0.00% | Laserson | 100.00% | 100.00% | 100.00% | High | Yes |
| 10 | Brust^11^ | Census | 100.00% | 100.00% | 100.00% | 24.10% | Laserson | 99.30% | 57.80% | 98.50% | Moderate | No |
| 11 | Cegielski^12,13^ | Census | 92.80% | 92.20% | 60.10% | 19.80% | Laserson | 100.00% | 68.30% | 98.20% | High | Yes |
| 12 | Chan (Denver) ^14^ | Census | 100.00% | 100.00% | 100.00% | 26.70% | Laserson | 100.00% | 80.00% | 100.00% | High | Yes |
| 13 | Dheda^15-17^ | Census | 100.00% | 100.00% | 61.50% | 4.70% | Laserson | 99.10% | 100.00% | 93.50% | High | Yes |
| 14 | Fox^18^ | Census | 93.10% | 96.60% | 100.00% | 3.40% | WHO2013 | 100.00% | 100.00% | 100.00% | High | Yes |
| 15 | Gegia^19^ | Census | 100.00% | 100.00% | 100.00% | 21.80% | Laserson | 100.00% | 72.90% | 100.00% | High | No |
| 16 | Guglielmetti^20,21^ | Census | 100.00% | 100.00% | 100.00% | 11.10% | WHO2013 | 100.00% | 100.00% | 100.00% | High | Yes |
| 17 | Guglielmetti^22^ | Census | 100.00% | 100.00% | 100.00% | 10% | WHO2013 | 100.00% | 100.00% | 90.00% | High | Yes |
| 18 | Hughes^23^ | Census | 94.90% | 94.90% | 100.00% | 25.40% | Laserson | 100.00% | 100.00% | 100.00% | High | No |
| 19 | Isaakidis^23,24^ | Census | 96.70% | 95.40% | 100.00% | 11.80% | Laserson | 100.00% | 100.00% | 98.00% | High | Yes |
| 20 | Jarlsberg^25^ | Census | 96.40% | 96.40% | 100.00% | 3.60% | Laserson | 100.00% | 92.90% | 100.00% | High | Yes |
| 21 | Kempker^26^ | Census | 100.00% | 100.00% | 94.90% | 32.70% | Laserson | 100.00% | 94.70% | 100.00% | High | Yes |
| 22 | Koenig^27^ | Census | 96.30% | 93.30% | 100.00% | 6.10% | Laserson | 99.40% | 100.00% | 100.00% | High | Yes |
| 23 | Koh^28,29^ | Census | 100.00% | 100.00% | 100.00% | 13.40% | WHO2013 | 100.00% | 100.00% | 100.00% | High | Yes |
| 24 | Kuksa^30^ | Census | 100.00% | 100.00% | 100.00% | 15% | Laserson | 100.00% | 100.00% | 100.00% | High | Yes |
| 25 | Kvasnovsky^31,32^ | Census | 100.00% | 100.00% | 100.00% | 11.50% | Laserson | 100.00% | 96.90% | 100.00% | High | Yes |
| 26 | Lange^33^ | Census | 94.00% | 96.70% | 100.00% | 20.10% | Laserson | 100.00% | 99.50% | 98.40% | High | Yes |
| 27 | Laniado-Laborin^34^ | Census | 100.00% | 100.00% | 100.00% | 13.50% | Laserson | 100.00% | 100.00% | 100.00% | High | Yes |
| 28 | Leung^35,36^ | Census | 100.00% | 100.00% | 100.00% | 19.90% | Laserson | 100.00% | 100.00% | 100.00% | High | Yes |
| 29 | Marks^37^ | Random | 92.30% | 91.50% | 100.00% | 12.30% | Neither | 100.00% | 85.40% | 100.00% | High | Yes |
| 30 | Migliori^38,39^ | Census | 96.60% | 96.60% | Unclear | 10.90% | WHO2013 | 100.00% | 98.10% | 99.30% | High | Yes |
| 31 | Migliori (BDQ)^40^ | Census | 97.00% | 100.00% | Unclear | 3.70% | WHO2013 | 100.00% | 99.30% | 100.00% | High | Yes |
| 32 | Milanov^41^ | Census | 94.00% | 94.00% | 100.00% | 2.00% | Laserson | 100.00% | 100.00% | 100.00% | High | Yes |
| 33 | Ndjeka^42^ | Unclear | 78.20% | 81.20% | Unclear | 21.10% | Laserson | 100.00% | 95.50% | 0.00% | Low | Yes |
| 34 | Ndjeka^43^ | Census | 100.00% | 100.00% | 100.00% | 18.50% | Both | 100.00% | 100.00% | 100.00% | Low | Yes |
| 35 | O’Donnell^44^ | Census | 100.00% | 100.00% | 100.00% | 13.20% | Laserson | 100.00% | 93.90% | 93.90% | High | Yes |
| 36 | Palmero^45^ | Census | 100.00% | 100.00% | 100.00% | 22.20% | WHO2013 | 100.00% | 100.00% | 100.00% | High | No |
| 37 | Podewils^46^ | Census | 91.00% | 91.20% | 100.00% | 15.20% | Laserson | 100.00% | 55.60% | 100.00% | High | Yes |
| 38 | Riekstina/Leimane^47^ | Census | 100.00% | 100.00% | 100.00% | 14.70% | Laserson | 100.00% | 94.00% | 100.00% | High | Yes |
| 39 | Rodrigues^48^ | Census | 87.00% | 85.00% | 100.00% | 10.00% | Laserson | 100.00% | 98.00% | 100.00% | High | Yes |
| 40 | Seo^49^ | Census | 100.00% | 100.00% | 100.00% | 16.00% | Laserson | 100.00% | 100.00% | 100.00% | High | Yes |
| 41 | Seung^50^ | Census | 80.20% | 80.20% | 100.00% | 1.40% | Unclear | 100.00% | 0% | 88.70% | High | No |
| 42 | Shim^29,51^ | Census | 100.00% | 100.00% | 86.40% | 8.20% | WHO2013 | 100.00% | 40.00% | 100.00% | High | Yes |
| 43 | Singla^52^ | Census | 100.00% | 100.00% | 100.00% | 13.80% | Laserson | 100.00% | 100.00% | 100.00% | High | Yes |
| 44 | Skrahina^53^ | Census | 100.00% | 100.00% | 100.00% | 1.00% | WHO2013 | 100.00% | 99.00% | 100.00% | High | Yes |
| 45 | Smith^54^ | Census | 100.00% | 100.00% | 100.00% | 21.50% | Laserson | 100.00% | 100.00% | 98.50% | High | Yes |
| 46 | TMC207-C208^55,56^ | RCT | 84.80% | 84.80% | 82.50% | 28.80% | Laserson | 100.00% | 100.00% | 100.00% | High | Yes |
| 47 | TMC207-C209^57^ | Census | 76.10% | 76.10% | 93.10% | 15.20% | Laserson | 100.00% | 96.50% | 100.00% | Moderate | Yes |
| 48 | Udwadia^58^ | Census | 100.00% | 100.00% | 100.00% | 27.80% | Laserson | 100.00% | 44.40% | 100.00% | High | Yes |
| 49 | van der Werf^59^ | Census | 100.00% | 98.20% | 100.00% | 13.40% | Laserson | 100.00% | 92.00% | 96.40% | High | Yes |
| 50 | Vasilyeva^60^ | Census | 94.40% | 94.40% | 100.00% | 16.00% | WHO2013 | 100.00% | 100.00% | 100.00% | High | Yes |
| 51 | Viiklepp^61^ | Census | 100.00% | 100.00% | 100.00% | 11.70% | Laserson | 100.00% | 99.70% | 100.00% | High | Yes |
| 52 | Yim/Kwak^62^ | Census | 100.00% | 100.00% | 100.00% | 4.90% | WHO2013 | 100.00% | 100.00% | 100.00% | High | Yes |
| 53 | Achar^63^ | Census | 66.10% | 65.30% | 100.00% | 22.00% | WHO2013 | 100.00% | 80.60% | 100.00% | Moderate | Yes |
| 54 | Isaakidis^64^ | Census | 95.00% | 96.00% | 100.00% | 6.00% | Laserson | 100.00% | 100.00% | 100.00% | High | Yes |
| 55 | Skrahina^65^ | Census | 96.40% | 95.50% | 100.00% | 0.00% | WHO2013 | 100.00% | 99.10% | 99.10% | High | Yes |
| SLI: Second-line injectable. FQ: fluoroquinolone. Both: indicates Laserson and WHO 2013 were used. Neither: indicates neither Laserson nor WHO 2013 were used. Quality Assessment Reference: Lancet 2018; 392: 821–34. | | | | | | | | | | | | |

# Supplement 2. Completeness of information, quality of all studies in the IPD, and their inclusion status in this analysis.

# Supplement 3. Detail on models for ecological level analysis.

To estimate conditional regression coefficients and their 95% CI for associations with site-level treatment duration a multivariable linear regression model was constructed that included the site-level proportion of female sex, HIV infection, extensive disease (defined as yes if AFB smear positive at baseline or if AFB smear status was missing, presence of radiographic findings of cavitation or bilateral disease), past first-line drug use, past second-line drug use, MDR-TB, MDR-TB plus resistance to FQ but SLI sensitive (MDR-FQ), MDR-TB plus resistance to SLI but FQ sensitive (MDR-SLI), MDR-TB plus resistance to both FQ and SLI (MDR-FQ+SLI), resistance to pyrazinamide, and proportion of patients at that site who received bedaquiline or linezolid. We also included the site-level mean number of effective drugs used, mean patient age, number of patients treated, and 2018 World Bank income category of the site.

# Supplement 4. Detail on descriptions and definitions of baseline patient characteristics, drug susceptibility testing, and treatments used.

Categorical baseline characteristics were described as n (%), and included the following: sex (male or female); body mass index category (underweight: <18.5 kg/m²; normal: ≥18.5 and <25 kg/m²; and overweight or obese: ≥25 kg/m); World Bank 2018 category of country level income (low/lower-middle, upper-middle, and high income); smoking (yes/no); alcohol use disorder (yes/no); HIV status (positive/negative); if HIV positive, on ART (yes/no); diabetes (yes/no); cavitation on chest radiography (yes/no); bilateral disease on chest radiography (yes/no); acid-fast bacilli (AFB) smear positivity at baseline (positive/negative); extensive disease (defined as yes if AFB smear positive at baseline or if AFB smear status was missing, presence of radiographic findings of cavitation or bilateral disease); past TB treatment, past first-line drug use, and past second-line drug use (all yes/no); resistance on drug susceptibility testing (DST) for FQ, SLI, linezolid, pyrazinamide, clofazimine, and cycloserine/terizidone (all yes/no); drug resistant profile category defined as only MDR-TB, MDR-TB plus resistance to FQ but SLI sensitive (MDR-FQ), MDR-TB plus resistance to SLI but FQ sensitive (MDR-SLI), MDR-TB plus resistance to both FQ and SLI (MDR-FQ+SLI); and each individual drug used during treatment (yes/no). All missing observations were included as a missing category for all categorical variables for descriptive statistics. Age, body mass index, deviation in treatment duration, and total individual duration of treatment were described using mean (SD), while total number of drugs, effective drugs (based on DST results), and new/limited access drugs used (bedaquiline, clofazimine, linezolid, and/or meropenem/imipenem) were described using median [IQR].

# Supplement 5. Detail on multivariate imputation by chained equations (MICE).

Missing data were imputed using age, sex, body mass index, previous treatment history, radiographic features, World Bank income level, drug susceptibility testing (DST) results for: fluoroquinolones (FQ), second line injectables (SLI), ethambutol, pyrazinamide, prothionamide/ethionamide, and para-aminosalicylic acid (PAS), as well as deviation in treatment duration. The deviation in treatment duration was imputed for those with either only planned or missing deviation in treatment duration for our sensitivity analyses, along with the other variables, however we only included subjects with non-missing duration in our primary analysis

# Supplement 6. Detail on inverse probability of selection weights.

We explored the possible effect of selection bias on our population by analyzing our final model adjusted with inverse probability of selection weights (IPSW) calculated using a logistic regression model with binary variable for inclusion (1: indicating inclusion; 0:indicating exclusion from study population) as the outcome and adjusted for age, sex, body mass index, World Bank income category, HIV status, diabetes, past first and second line drug use, radiographic findings, drug resistance to FQ, SLIs, ethambutol, PAS, amikacin/kanamycin/capreomycin/streptomycin, ethionamide/prothionamide and pyrazinamide, as well as number of drugs and use of bedaquiline, pyrazinamide, FQ, linezolid, clofazimine, and cycloserine/terizidone.

# Supplement 7. Detailed description of E-Values.

With E-values, linear regression coefficients are converted to an approximation of the risk ratio from an approximation of the odds ratio as defined in Chinn^1^ and VanderWeele^2^, using the following formula: RR = $\sqrt{e^{(\frac{\beta}{SDo}*1.81)}}$ where β is the regression coefficient of an exposure and SD_o_ is the standard deviation of our treatment duration outcome. The E-values are calculated as follows: for RR>1: E-Value = RR + $\sqrt{RR*(RR-1)}$ with the confidence interval (CI) of the E-value being the lower limit of the RR (LL) + $\sqrt{LL*(LL-1)}$ , if the LL>1, while if LL≤ then the CI = 1; for RR<1: E-value = 1/RR + $\sqrt{1/RR*([1/RR]-1)}$ with the CI of the E-value being 1/upper limit of the RR (UL) +$\sqrt{1/UL*([1/UL]-1)}$ , if the UL<1, while if UL≥ 1 then CI = 1.

1. Chinn S. A simple method for converting an odds ratio to effect size for use in meta-analysis. *Stat Med* 2000; **19**(22): 3127-31.

2. VanderWeele TJ. On a Square-Root Transformation of the Odds Ratio for a Common Outcome. *Epidemiology* 2017; **28**(6): e58-e60.

# Supplement 8. Detail on variable selection due to correlation in the multivariable model for the primary analysis.

Pearson coefficients were used to assess correlation between variables to be included. When highly correlated variables were present, we chose the more clinically relevant variable. This occurred between the following: individual DST results and MDR categories; number of drugs used, and number of effective drugs used; and extensive disease and individual measures of disease extent (AFB smear, cavitation/bilateral disease on x-ray). We included MDR categories as they capture the same information as DST results but with more clinically relevant categorization. Number of drugs was included as number of effective drugs was correlated with use of bedaquiline and clofazimine. Finally, the individual measures of disease extent were used as these provided a more granular description of the markers of extensive disease. The final covariate list included: age (continuous), sex, body mass index (continuous), HIV infection, AFB smear, cavitation on x-ray, bilateral disease on x-ray, past first- and second-line drug use, MDR category, number of drugs used in treatment, and use of bedaquiline, clofazimine, PAS, moxifloxacin, linezolid, kanamycin, amoxicillin-clavulanate (Amx-Clv), capreomycin, and clarithromycin at anytime during treatment.

# Supplemental Table S1. Comparison of patients in the IPD who were included and excluded from this analysis.

|  | **Excluded patients (unsuccessful treatment)** | **Exclude patients (successful treatment)** | **Included patients** | **Overall** |
| --- | --- | --- | --- | --- |
|  | **5387** | **1183** | **6702** | **13272** |
|  | N (%) |  | N (%) | N (%) |
| Sex (Female) | 2009 (37.3) | 481 (40.7) | 2719 (40.6) | 5209 (39.3) |
| Age (mean (SD)) | 37.16 (12.65) | 35.51 (12.68) | 37.02 (12.96) | 36.94 (12.81) |
| Body mass index (mean (SD)) | 19.46 (3.74) | 20.25 (3.48) | 20.47 (3.84) | 20.07 (3.81) |
| Body mass index category (%) |  |  |  |  |
| Normal | 1183 (22.0) | 268 (22.7) | 2024 (30.2) | 3475 (26.2) |
| Underweight | 982 (18.2) | 134 (11.3) | 1028 (15.3) | 2144 (16.2) |
| Overweight/Obese | 160 (3.0) | 39 (3.3) | 377 (5.6) | 576 (4.3) |
| Missing | 3062 (56.8) | 742 (62.7) | 3273 (48.8) | 7077 (53.3) |
| 2018 World Bank income category |  |  |  |  |
| Low/Low-middle | 1043 (19.4) | 596 (50.4) | 1226 (18.3) | 2865 (21.6) |
| Upper-Middle | 3635 (67.5) | 562 (47.5) | 3555 (53.0) | 7752 (58.4) |
| High | 709 (13.2) | 25 (2.1) | 1921 (28.7) | 2655 (20.0) |
| Smoking (%) |  |  |  |  |
| Ex-smoker or never smoker | 1070 (19.9) | 241 (20.4) | 1834 (27.4) | 3145 (23.7) |
| Current smoker | 750 (13.9) | 105 (8.9) | 939 (14.0) | 1794 (13.5) |
| Unknown | 3567 (66.2) | 837 (70.8) | 3929 (58.6) | 8333 (62.8) |
| HIV (%) |  |  |  |  |
| Negative | 3005 (55.8) | 821 (69.4) | 4771 (71.2) | 8597 (64.8) |
| Positive | 1969 (36.6) | 152 (12.8) | 1859 (27.7) | 3980 (30.0) |
| Unknown | 413 (7.7) | 210 (17.8) | 72 (1.1) | 695 (5.2) |
| If HIV positive, on ART | 1375 (69.8) | 16 (10.5) | 1686 (90.7) | 3077 (77.3) |
| Diabetes (%) |  |  |  |  |
| No | 2245 (41.7) | 339 (28.7) | 3311 (49.4) | 5895 (44.4) |
| Yes | 254 (4.7) | 34 (2.9) | 466 (7.0) | 754 (5.7) |
| Unknown | 2888 (53.6) | 810 (68.5) | 2925 (43.6) | 6623 (49.9) |
| Past TB treatment (%) |  |  |  |  |
| No | 1297 (24.1) | 175 (14.8) | 2336 (34.9) | 3808 (28.7) |
| Yes | 3986 (74.0) | 969 (81.9) | 4271 (63.7) | 9226 (69.5) |
| Unknown | 104 (1.9) | 39 (3.3) | 95 (1.4) | 238 (1.8) |
| Past first-line TB drug use (%) |  |  |  |  |
| No | 1297 (24.1) | 175 (14.8) | 2336 (34.9) | 3808 (28.7) |
| Yes | 3986 (74.0) | 969 (81.9) | 4271 (63.7) | 9226 (69.5) |
| Unknown | 104 (1.9) | 39 (3.3) | 95 (1.4) | 238 (1.8) |
| Past second- line TB drug used (%) |  |  |  |  |
| No | 3377 (62.7) | 662 (56.0) | 5048 (75.3) | 9087 (68.5) |
| Yes | 1141 (21.2) | 54 (4.6) | 1226 (18.3) | 2421 (18.2) |
| Unknown | 869 (16.1) | 467 (39.5) | 428 (6.4) | 1764 (13.3) |
| Cavitation on X-ray (%) |  |  |  |  |
| No | 807 (15.0) | 159 (13.4) | 1606 (24.0) | 2572 (19.4) |
| Yes | 1618 (30.0) | 211 (17.8) | 2308 (34.4) | 4137 (31.2) |
| Unknown | 2962 (55.0) | 813 (68.7) | 2788 (41.6) | 6563 (49.4) |
| Bilateral disease (%) |  |  |  |  |
| No | 488 (9.1) | 174 (14.7) | 1122 (16.7) | 1784 (13.4) |
| Yes | 1526 (28.3) | 262 (22.1) | 1999 (29.8) | 3787 (28.5) |
| Unknown | 3373 (62.6) | 747 (63.1) | 3581 (53.4) | 7701 (58.0) |
| AFB smear result (%) |  |  |  |  |
| Negative | 1049 (19.5) | 36 (3.0) | 1974 (29.5) | 3059 (23.0) |
| Positive | 3028 (56.2) | 163 (13.8) | 4280 (63.9) | 7471 (56.3) |
| Unknown | 1310 (24.3) | 984 (83.2) | 448 (6.7) | 2742 (20.7) |
| Extensive disease, yes (%) | 3360 (62.4) | 415 (35.1) | 4512 (67.3) | 8287 (62.4) |
| DST Performed for Fluoroquinolone | 5205 (96.6) | 1131 (95.6) | 6449 (96.2) | 12785 (96.3) |
| If DST Performed, Fluoroquinolone Resistant | 1293 (24.8) | 149 (13.2) | 1172 (18.2) | 2614 (20.4) |
| DST Performed for Second Line Injectables | 5212 (96.8) | 1130 (95.5) | 6455 (96.3) | 12797 (96.4) |
| If DST Performed, Second Line Injectable Resistant | 1599 (30.7) | 131 (11.6) | 1629 (25.2) | 3359 (26.2) |
| DST Performed for Linezolid | 250 (4.6) | 23 (1.9) | 665 (9.9) | 938 (7.1) |
| If DST Performed, Linezolid Resistant | 12 (4.8) | 0 (0.0) | 16 (2.4) | 28 (3.0) |
| DST Performed for Pyrazinamide | 1760 (32.7) | 440 (37.2) | 3490 (52.1) | 5690 (42.9) |
| If DST Performed, Pyrazinamide Resistant | 1019 (57.9) | 237 (53.9) | 1859 (53.3) | 3115 (54.7) |
| DST Performed for Clofazimine | 104 (1.9) | 11 (0.9) | 252 (3.8) | 367 (2.8) |
| If DST Performed, Clofazimine Resistant | 2 (1.9) | 1 (9.1) | 9 (3.6) | 12 (3.3) |
| DST Performed for Cycloserine/Terizidone | 1863 (34.6) | 956 (80.8) | 2034 (30.3) | 4853 (36.6) |
| If DST Performed, Cycloserine/Terizidone Resistant | 136 (7.3) | 34 (3.6) | 260 (12.8) | 430 (8.9) |
| MDR category (%) |  |  |  |  |
| MDR/RR-TB FQ &SLI sensitive | 3198 (59.4) | 893 (75.5) | 4337 (64.7) | 8428 (63.5) |
| MDR/RR-TB + FQ resistant & SLI sensitive | 710 (13.2) | 89 (7.5) | 929 (13.9) | 1728 (13.0) |
| MDR/RR-TB + SLI resistant & FQ sensitive | 404 (7.5) | 106 (9.0) | 475 (7.1) | 985 (7.4) |
| MDR/RR-TB + SLI & FQ resistance | 888 (16.5) | 42 (3.6) | 688 (10.3) | 1618 (12.2) |
| No DST | 187 (3.5) | 53 (4.5) | 273 (4.1) | 513 (3.9) |
| MDR/RR-TB + SLI & FQ resistance vs. all others (%) |  |  |  |  |
| No | 4312 (80.0) | 1088 (92.0) | 5741 (85.7) | 11141 (83.9) |
| Yes | 888 (16.5) | 42 (3.6) | 688 (10.3) | 1618 (12.2) |
| U | 187 (3.5) | 53 (4.5) | 273 (4.1) | 513 (3.9) |
| Used Ethambutol Ever During Treatment = Yes (%) | 2490 (46.2) | 281 (23.8) | 2895 (43.2) | 5666 (42.7) |
| Used Pyrazinamide Ever During Treatment = Yes (%) | 4686 (87.0) | 1156 (97.7) | 5175 (77.2) | 11017 (83.0) |
| Used Streptomycin Ever During Treatment = Yes (%) | 386 (7.2) | 2 (0.2) | 692 (10.3) | 1080 (8.1) |
| Used Rifabutin Ever During Treatment = Yes (%) | 58 (1.1) | 1 (0.1) | 154 (2.3) | 213 (1.6) |
| Used Amikacin Ever During Treatment = Yes (%) | 621 (11.5) | 168 (14.2) | 1048 (15.6) | 1837 (13.8) |
| Used Capreomycin Ever During Treatment = Yes (%) | 1569 (29.1) | 232 (19.6) | 1446 (21.6) | 3247 (24.5) |
| Used Kanamycin Ever During Treatment = Yes (%) | 2787 (51.7) | 784 (66.3) | 3151 (47.0) | 6722 (50.6) |
| Used Ofloxacin Ever During Treatment = Yes (%) | 1658 (30.8) | 528 (44.6) | 1373 (20.5) | 3559 (26.8) |
| Used Ciprofloxacin Ever During Treatment = Yes (%) | 204 (3.8) | 4 (0.3) | 266 (4.0) | 474 (3.6) |
| Used Moxifloxacin Ever During Treatment = Yes (%) | 2194 (40.7) | 109 (9.2) | 3459 (51.6) | 5762 (43.4) |
| Used Levofloxacin Ever During Treatment = Yes (%) | 1123 (20.8) | 535 (45.2) | 1889 (28.2) | 3547 (26.7) |
| Used Ethionamide Ever During Treatment = Yes (%) | 3111 (57.8) | 676 (57.1) | 2859 (42.7) | 6646 (50.1) |
| Used Prothionamide Ever During Treatment = Yes (%) | 1453 (27.0) | 463 (39.1) | 2258 (33.7) | 4174 (31.4) |
| Used Cycloserine Ever During Treatment = Yes (%) | 2351 (43.6) | 899 (76.0) | 2873 (42.9) | 6123 (46.1) |
| Used Terizidone Ever During Treatment = Yes (%) | 1963 (36.4) | 29 (2.5) | 2922 (43.6) | 4914 (37.0) |
| Used PAS Ever During Treatment = Yes (%) | 2344 (43.5) | 565 (47.8) | 2759 (41.2) | 5668 (42.7) |
| Used Linezolid Ever During Treatment = Yes (%) | 638 (11.8) | 52 (4.4) | 1594 (23.8) | 2284 (17.2) |
| Used Clofazimine Ever During Treatment = Yes (%) | 651 (12.1) | 111 (9.4) | 1101 (16.4) | 1863 (14.0) |
| Used Amx-Clv Ever During Treatment = Yes (%) | 763 (14.2) | 93 (7.9) | 994 (14.8) | 1850 (13.9) |
| Used Thioacetazone Ever During Treatment = Yes (%) | 30 (0.6) | 0 (0.0) | 68 (1.0) | 98 (0.7) |
| Used Clarithromycin Ever During Treatment = Yes (%) | 507 (9.4) | 84 (7.1) | 485 (7.2) | 1076 (8.1) |
| Used Imipenem-Cilastatin Ever During Treatment = Yes (%) | 78 (1.4) | 2 (0.2) | 237 (3.5) | 317 (2.4) |
| Used Meropenem Ever During Treatment = Yes (%) | 23 (0.4) | 3 (0.3) | 61 (0.9) | 87 (0.7) |
| Used Bedaquiline Ever During Treatment = Yes (%) | 756 (14.0) | 17 (1.4) | 1605 (23.9) | 2378 (17.9) |
| Used Delamanid Ever During Treatment = Yes (%) | 46 (0.9) | 0 (0.0) | 114 (1.7) | 160 (1.2) |
| Number of drugs (median [IQR]) | 5 [4, 5] | 4 [4, 5] | 5.0 [4.0, 6.0] | 5.00 [4.00, 5.00] |
| Number of effective drugs (median [IQR]) | 4 [4, 5] | 4 [4, 5] | 4.0 [4.0, 5.0] | 4.00 [4.00, 5.00] |
| Number of limited access drugs** (median [IQR]) | 0 [0, 0] | 0 [0, 0] | 0.0 [0.0, 1.0] | 0.00 [0.00, 1.00] |
| Total Treatment duration (mean (SD)) | 14.45 (9.81) | NA | 22.0 (4.6) | 19.00 (7.97) |
| Median [IQR] | 13.9 [5.8, 23] | NA | 22 [19, 24] | 21 [16, 24] |
| SD: standard deviation; XDR: extensively drug resistant tuberculosis; MDR: multidrug resistant tuberculosis; TB: tuberculosis; AFB: acid-fast bacillus; Amx-Clv: Amoxicillin-Clavulinic Acid;  .** includes bedaquiline, clofazimine, linezolid, imipenem, and meropenem. | | | | |

# Supplemental Table S2. Mean total treatment duration and total number of patients at each site included in the study population.

| **Author** | **Treatment site*** | **Number of patients** | **Mean total treatment duration (SD)** |
| --- | --- | --- | --- |
| Ahuja | USA | 38 | 23.5 (8.9) |
| Anderson | UK | 90 | 21.4 (7.2) |
| Fox | Australia | 25 | 21.7 (3.7) |
| Bang | Denmark | 19 | 18.3 (4.1) |
| Barry/Flood (Calif) | USA | 45 | 21.1 (3.4) |
| Barry (Korea) | South Korea | 30 | 25.1 (3.1) |
| TMC207-C208 | Brazil | 2 | 22 (3.8) |
|  | India | 4 | 24 (0) |
|  | Latvia | 3 | 21.8 (2) |
|  | Peru | 17 | 21 (2.6) |
|  | Philippines | 1 | 25.6 (NA) |
|  | South Africa | 28 | 22.5 (3.2) |
|  | Thailand | 1 | 16.3 (NA) |
| Skrahina | Belarus | 94 | 23.6 (1.7) |
| Skrahina (2019) | Belarus | 106 | 23.2 (2.7) |
| Bonnet | Georgia | 68 | 26.7 (5.3) |
| Rodrigues | Brazil | 82 | 18.4 (2.2) |
| Brode | Canada | 17 | 24.6 (1.9) |
| Cegielski | Estonia | 30 | 21.6 (3.6) |
|  | Latvia | 99 | 20.3 (3.6) |
|  | Peru | 109 | 23.8 (6.8) |
|  | Philippines | 320 | 21.6 (3.3) |
|  | Russia | 81 | 21.9 (3.5) |
|  | South Africa | 250 | 24.1 (4) |
|  | South Korea | 46 | 25.8 (6) |
|  | Taiwan | 45 | 21.4 (2.1) |
|  | Thailand | 41 | 20.3 (3.8) |
| Chan (Denver) | USA | 7 | 28.1 (17.1) |
| Dheda | South Africa | 14 | 33.9 (12.1) |
| Guglielmetti | France | 35 | 21.6 (3.2) |
| Guglielmetti | France | 9 | 25.1 (2) |
| Isaakidis | India | 69 | 23 (6.5) |
| TMC207-209 | Asia | 58 | 23.1 (2.9) |
|  | Europe | 38 | 20.1 (2.9) |
|  | Peru | 10 | 18.2 (2.5) |
|  | South Africa | 39 | 22.3 (3.3) |
| Jarlsberg | USA | 20 | 21.6 (4.5) |
| Kempker | Georgia | 84 | 24.1 (3.2) |
| Koenig | Haiti | 126 | 24 (0.6) |
| Koh | South Korea | 272 | 23 (5.4) |
| Kvasnovsky | South Africa | 34 | 29.7 (7.1) |
| Lange | Germany | 91 | 23.8 (1.5) |
| Laniado-Laborin | Mexico | 37 | 22.6 (5.3) |
| Kuksa | Latvia | 31 | 18.4 (3.6) |
| Barkane | Latvia | 26 | 17 (4) |
| Leung | Hong Kong | 136 | 16.7 (3) |
| Marks | USA | 92 | 22.6 (6) |
| Migliori | Belarus | 6 | 16.8 (2.8) |
|  | Belgium | 13 | 25.3 (5.3) |
|  | Brazil | 3 | 35.3 (11) |
|  | Ecuador | 1 | 12 (NA) |
|  | Greece | 11 | 23 (5.4) |
|  | Italy | 74 | 19.9 (3.6) |
|  | Netherlands | 43 | 17.5 (3.5) |
|  | Peru | 13 | 20 (11.9) |
|  | Slovakia | 2 | 25 (1.4) |
|  | UK | 3 | 21.8 (3.9) |
| Migliori (BDQ) | Australia | 1 | 24 (NA) |
|  | Belgium | 2 | 21.3 (0.4) |
|  | Greece | 2 | 25 (0) |
|  | India | 11 | 34.3 (4.5) |
|  | Italy | 7 | 22.6 (2.8) |
|  | Netherlands | 2 | 20 (0) |
|  | Peru | 1 | 36 (NA) |
|  | Russia | 48 | 23.4 (5) |
|  | South Africa | 24 | 24.7 (4.7) |
|  | Sweden | 3 | 26.7 (6.4) |
| Milanov | Bulgaria | 23 | 23.4 (2.1) |
| Achar | Uzbekistan | 63 | 26.4 (4.4) |
| Isaakidis | India | 63 | 23 (3.9) |
| Ndjeka | South Africa | 60 | 20.4 (2.8) |
| O’Donnell | South Africa | 25 | 24.1 (2.1) |
| Podewils | Philippines | 385 | 20.7 (3) |
| Riekstina/Leimane | Latvia | 108 | 19.4 (3.4) |
| Vasilyeva | Russia | 68 | 20 (3.4) |
| Shim | South Korea | 42 | 22.3 (4.5) |
| Singla | India | 20 | 24.7 (0.8) |
| Smith | Russia | 114 | 26.6 (3.9) |
| Ndjeka | South Africa | 2128 | 21.7 (3.2) |
| Seo | South Korea | 19 | 30 (11.1) |
| Udwadia | India | 12 | 19 (2.3) |
| van der Werf | Netherlands | 75 | 16.7 (3.4) |
| Viiklepp | Estonia | 205 | 19.3 (4.3) |
| Yim/Kwak | South Korea | 103 | 25.3 (7.5) |
| Treatment sites were identified by country within a given study. SD: standard deviation | | | |

# Supplemental Table S3. Associations between deviation in treatment duration from site mean and patient characteristics, resistance categories, and drugs used from the full multivariable model with inverse probability of selection weights and without (primary analysis), including all variables listed.

|  | **With inverse probability of selection weighting** | **Without weighting** |
| --- | --- | --- |
|  | **months (95% CI)** | **months (95% CI)** |
| **Clinical characteristics** |  |  |
| Age (per year increase) | 0 (0.0, 0.01) | 0 (0.0, 0.01) |
| Sex (Female) | -0.09 (-0.29, 0.11) | -0.11 (-0.3, 0.08) |
| Body mass index (per unit increase) | -0.02 (-0.06, 0.01) | -0.02 (-0.05, 0.01) |
| HIV infection | 0.34 (0.08, 0.61) | 0.33 (0.06, 0.6) |
| AFB smear positive | 0.77 (0.54, 1.0) | 0.82 (0.6, 1.04) |
| Cavitation on X-Ray | 0.35 (0.09, 0.61) | 0.3 (0.05, 0.56) |
| Bilateral disease on X-ray | 0.4 (0.1, 0.71) | 0.24 (-0.07, 0.54) |
| **Treatment history and drug resistance** | |  |
| Past first-line drug use | 0.36 (0.11, 0.62) | 0.38 (0.14, 0.62) |
| Past second-line drug use | 0.29 (-0.06, 0.65) | 0.21 (-0.16, 0.58) |
| Number of drugs used | 0.3 (0.16, 0.43) | 0.34 (0.21, 0.47) |
| MDR/RR-TB + FQ resistant & SLI sensitive | 0.39 (0.08, 0.71) | 0.34 (0.03, 0.65) |
| MDR/RR-TB + SLI resistant & FQ sensitive | 0.72 (0.3, 1.15) | 0.78 (0.36, 1.21) |
| MDR/RR-TB + SLI & FQ resistance | 0.63 (0.21, 1.04) | 0.61 (0.21, 1.02) |
| **Drugs used in treatment** |  |  |
| Used Bedaquiline Ever During Treatment | -0.39 (-0.79, 0.0) | -0.51 (-0.87, -0.15) |
| Used Linezolid Ever During Treatment | 0.09 (-0.3, 0.48) | 0.29 (-0.06, 0.63) |
| Used Moxifloxacin Ever During Treatment | 0.37 (0.13, 0.61) | 0.32 (0.09, 0.54) |
| Used Clofazimine Ever During Treatment | 0.16 (-0.24, 0.56) | -0.15 (-0.51, 0.21) |
| Used Capreomycin Ever During Treatment | 0.7 (0.41, 0.99) | 0.69 (0.41, 0.97) |
| Used Kanamycin Ever During Treatment | 0.46 (0.22, 0.71) | 0.44 (0.2, 0.68) |
| Used PAS Ever During Treatment | -0.05 (-0.3, 0.2) | -0.03 (-0.27, 0.21) |
| Used Amx-Clv Ever During Treatment | 0.67 (0.32, 1.02) | 0.73 (0.4, 1.06) |
| Used Clarithromycin Ever During Treatment | 0.85 (0.43, 1.28) | 1.12 (0.71, 1.53) |

# Supplemental Table S4. E-values for selected characteristics with largest effect estimates for deviation in treatment duration.

|  | **Linear regression estimate (95% CI)** | **Converted RR**  **(95% CI)** | **E-Value (limit†)** |
| --- | --- | --- | --- |
| Used Bedaquiline Ever During Treatment | -0.51 (-0.87, -0.15) | 0.89 (0.82, 0.97) | 1.50 (NE, 1.23) |
| Used Clarithromycin Ever During Treatment | 1.12 (0.71, 1.53) | 1.29 (1.18, 1.42) | 1.90 (1.63, NE) |
| MDR/RR-TB + SLI resistant & FQ sensitive | 0.34 (0.03, 0.65) | 1.08 (1.01, 1.16) | 1.38 (1.09, NE) |
| MDR/RR-TB + FQ resistant & SLI sensitive | 0.78 (0.36, 1.21) | 1.20 (1.09, 1.32) | 1.68 (1.39, NE) |
| Past first-line drug use | 0.38 (0.14, 0.62) | 1.09 (1.03, 1.15) | 1.41 (1.21, NE) |
| HIV infection | 0.33 (0.06, 0.60) | 1.08 (1.01, 1.15) | 1.37 (1.13, NE) |
| AFB smear positive | 0.82 (0.60, 1.04) | 1.21 (1.15, 1.27) | 1.71 (1.56, NE) |
| Cavitation on X-Ray | 0.30 (0.05, 0.56) | 1.07 (1.01, 1.14) | 1.35 (1.12, NE) |
| RR: risk ratio, CI: confidence interval. Risk ratios are converted from linear regression coefficient. †E-values are calculated as follows: for RR>1: E-Value = RR + √(RR*(RR-1)) with the confidence interval (CI) of the E-value being the lower limit of the RR (LL) + √(LL*(LL-1)) , if the LL>1, while if LL≤ then the CI = 1; for RR<1: E-value = 1/RR + √(1/RR*([1/RR]-1)) with the CI of the E-value being 1/upper limit of the RR (UL) +√(1/UL*([1/UL]-1)) , if the UL<1, while if UL≥ 1 then CI = 1. E-values interpreted as the minimum strength of association that an unmeasured confounder would need to have with both the treatment and the outcome to fully explain away a specific treatment-outcome association. | | | |

# Supplemental Table S5. Associations between deviation in treatment duration from site mean and patient characteristics, resistance categories, and drugs used by different combinations of subgroups, and their regression estimates and 95% confidence intervals (CI) from a multivariable linear mixed model including all variables listed (unless specified otherwise).

|  | **Those with extensive disease and only MDR/RR-TB** | **Those without extensive disease and with resistance in addition to**  **MDR/RR-TB*** | **Those with past treatment and only MDR/RR-TB** | **Those without past treatment and resistance in addition to MDR/RR-TB*** |
| --- | --- | --- | --- | --- |
| **Characteristic** | **months (95% CI)** | **months (95% CI)** | **months (95% CI)** | **months (95% CI)** |
| **Clinical characteristics** |  |  |  |  |
| Age (per year increase) | 0.01 (0, 0.02) | 0 (-0.02, 0.03) | 0.02 (0.01, 0.03) | -0.02 (-0.05, 0.01) |
| Sex (Female) | -0.21 (-0.48, 0.05) | 0.16 (-0.5, 0.82) | -0.18 (-0.47, 0.11) | -0.76 (-1.52, 0) |
| Body mass index (per unit increase) | -0.03 (-0.07, 0.01) | -0.02 (-0.13, 0.09) | -0.04 (-0.08, 0) | 0 (-0.14, 0.14) |
| HIV infection | 0.32 (-0.03, 0.67) | 0.42 (-0.38, 1.23) | 0.39 (-0.01, 0.79) | 0.07 (-0.97, 1.11) |
| AFB smear positive | NE | NE | 0.69 (0.34, 1.05) | 0.8 (-0.03, 1.63) |
| Cavitation on X-Ray | NE | NE | 0.25 (-0.11, 0.61) | 0.83 (-0.12, 1.79) |
| Bilateral disease on X-ray | NE | NE | 0.35 (-0.08, 0.78) | -0.05 (-1.15, 1.04) |
| **Treatment history** | |  |  |  |
| Past first-line drug use | 0.56 (0.24, 0.88) | 0.16 (-0.7, 1.02) | NE | NE |
| Past second-line drug use | 0.05 (-0.52, 0.63) | 0.41 (-0.46, 1.29) | NE | NE |
| Number of drugs used | 0.4 (0.21, 0.6) | 0.07 (-0.31, 0.45) | 0.29 (0.07, 0.5) | 0.3 (-0.17, 0.77) |
| **Drugs used in treatment** |  |  |  |  |
| Used Bedaquiline Ever During Treatment | -0.43 (-0.92, 0.05) | -0.43 (-1.42, 0.57) | -0.69 (-1.24, -0.13) | -1.41 (-2.71, -0.1) |
| Used Moxifloxacin Ever During Treatment | 0.52 (0.18, 0.85) | 0.06 (-0.6, 0.72) | 0.25 (-0.09, 0.6) | 0.79 (-0.04, 1.61) |
| Used Linezolid Ever During Treatment | 0.52 (-0.07, 1.12) | 0.9 (-0.04, 1.85) | 0.6 (-0.1, 1.29) | 1.5 (0.37, 2.63) |
| Used Clofazimine Ever During Treatment | -0.84 (-1.47, -0.22) | 0.42 (-0.5, 1.35) | -0.22 (-0.95, 0.5) | 0.57 (-0.57, 1.7) |
| Used Capreomycin Ever During Treatment | 0.74 (0.31, 1.16) | 0.89 (0.14, 1.65) | 0.71 (0.24, 1.17) | 0.98 (-0.04, 2) |
| Used Kanamycin Ever During Treatment | 0.49 (0.18, 0.8) | 1.12 (0.21, 2.03) | 0.24 (-0.09, 0.57) | 1.45 (0.41, 2.49) |
| Used Amx-Clv Ever During Treatment | 1.32 (0.73, 1.92) | 0.11 (-0.64, 0.85) | 1.42 (0.77, 2.06) | 0.12 (-0.89, 1.14) |
| Used Clarithromycin Ever During Treatment | 0.07 (-0.62, 0.76) | 1.74 (0.74, 2.75) | 0.86 (0.15, 1.57) | 1.57 (0.11, 3.03) |
| * Includes MDR/RR-TB + resistance to a fluoroquinolone (but not a second-line injectable), MDR/RR-TB + resistance to a second-line injectable (but not a fluoroquinolone), and MDR/RR-TB with resistance to both a fluoroquinolone and a second-line injectable. All models also adjusted for use of PAS. | | | | |

# Supplemental Figure S1. Changes in World Health Organization treatment guidelines for multidrug-resistant tuberculosis between 1997 and 2022.


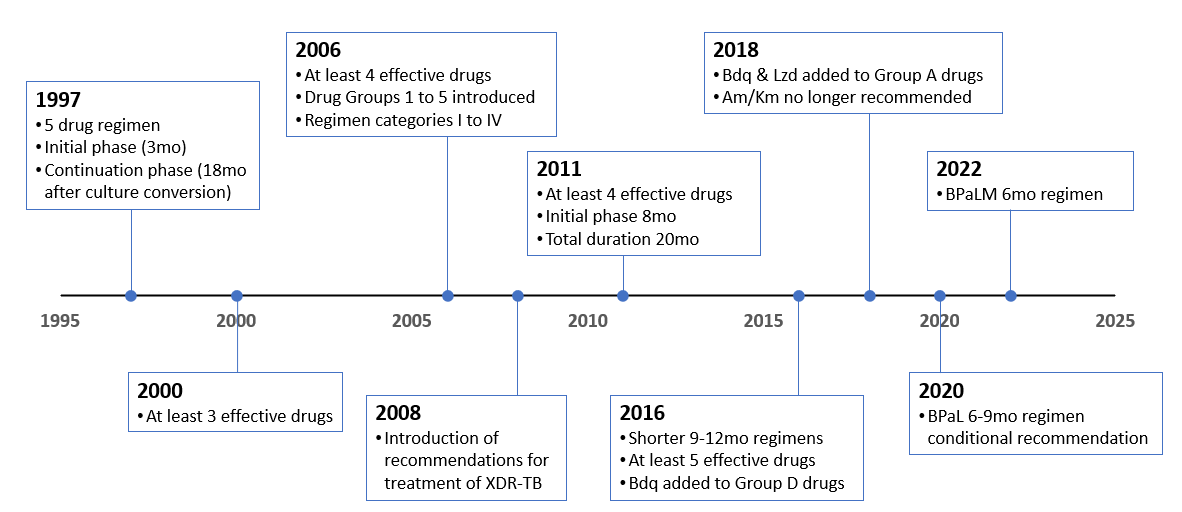


Abbreviations: mo: months; XDR-TB: extensively drug resistant tuberculosis; Bdq: bedaquiline; Lzd: linezolid; Am: amikacin; Km: kanamycin; BPaL: bedaquiline, pretomanid, and linezolid; BPaLM: bedaquiline, pretomanid, linezolid and moxifloxacin.

# Supplemental Figure S2. Forest plot of associations between deviation in treatment duration (in months) from site mean and patient characteristics, resistance categories, and drugs used analyzed using imputed outcomes for subjects with missing or planned duration. Estimates and 95% confidence intervals (CI) from regression using a multivariable linear mixed model, including all variables shown (n = 7885).


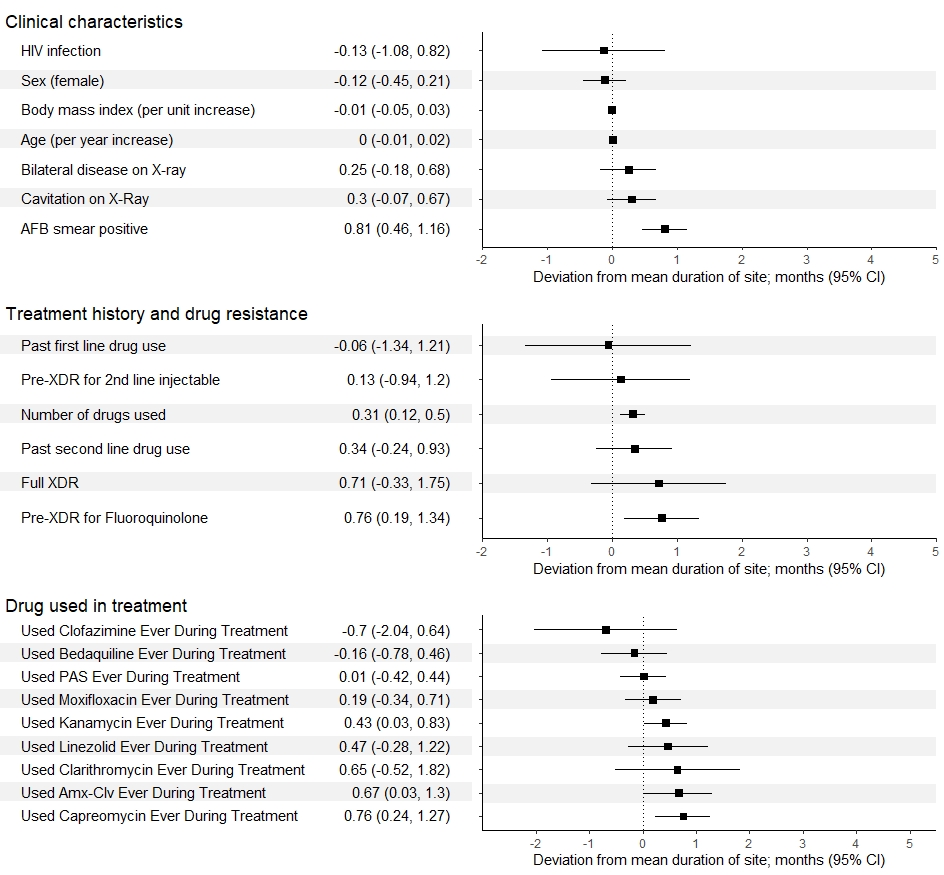


* Conditional R^2^ for model: 0.15. Note the patients whose outcomes were imputed include those who had either missing duration or only planned duration.

# References

1. Ahmad N, Javaid A, Basit A, et al. Management and treatment outcomes of MDR-TB: results from a setting with high rates of drug resistance. *Int J Tuberc Lung Dis* 2015; **19**(9): 1109-14, i-ii.

2. Anger HA, Dworkin F, Sharma S, Munsiff SS, Nilsen DM, Ahuja SD. Linezolid use for treatment of multidrug-resistant and extensively drug-resistant tuberculosis, New York City, 2000-06. *The Journal of antimicrobial chemotherapy* 2010; **65**(4): 775-83.

3. Anderson LF, Tamne S, Watson JP, et al. Treatment outcome of multi-drug resistant tuberculosis in the United Kingdom: retrospective-prospective cohort study from 2004 to 2007. *Euro surveillance : bulletin Europeen sur les maladies transmissibles = European communicable disease bulletin* 2013; **18**(40).

4. Bang D, Lillebaek T, Thomsen VO, Andersen AB. Multidrug-resistant tuberculosis: treatment outcome in Denmark, 1992-2007. *Scandinavian journal of infectious diseases* 2010; **42**(4): 288-93.

5. Barkane L. Unpublished data (Riga, Latvia). 2018.

6. Lee M, Cho SN, Barry CE, 3rd, Song T, Kim Y, Jeong I. Linezolid for XDR-TB--Final Study Outcomes. *N Engl J Med* 2015; **373**(3): 290-1.

7. Lee M, Lee J, Carroll MW, et al. Linezolid for treatment of chronic extensively drug-resistant tuberculosis. *N Engl J Med* 2012; **367**(16): 1508-18.

8. Barry PM, Flood J, Lowenthal P, Westenhouse J, California Department of Public Health. Unpublished data (California, USA). 2016.

9. Bonnet M, Pardini M, Meacci F, et al. Treatment of tuberculosis in a region with high drug resistance: outcomes, drug resistance amplification and re-infection. *PLoS One* 2011; **6**(8): e23081.

10. Brode S, West Park Healthcare Centre. Unpublished data (Toronto, Canada). 2016.

11. Brust JC, Gandhi NR, Carrara H, Osburn G, Padayatchi N. High treatment failure and default rates for patients with multidrug-resistant tuberculosis in KwaZulu-Natal, South Africa, 2000-2003. *Int J Tuberc Lung Dis* 2010; **14**(4): 413-9.

12. Cegielski JP, Kurbatova E, van der Walt M, et al. Multidrug-Resistant Tuberculosis Treatment Outcomes in Relation to Treatment and Initial Versus Acquired Second-Line Drug Resistance. *Clin Infect Dis* 2016; **62**(4): 418-30.

13. Yuen CM, Kurbatova EV, Tupasi T, et al. Association between Regimen Composition and Treatment Response in Patients with Multidrug-Resistant Tuberculosis: A Prospective Cohort Study. *PLoS medicine* 2015; **12**(12): e1001932.

14. Chan ED, National Jewish Health. Unpublished data (Denver, USA). 2016.

15. Pietersen E, Ignatius E, Streicher EM, et al. Long-term outcomes of patients with extensively drug-resistant tuberculosis in South Africa: a cohort study. *Lancet* 2014; **383**(9924): 1230-9.

16. Shean K, Streicher E, Pieterson E, et al. Drug-associated adverse events and their relationship with outcomes in patients receiving treatment for extensively drug-resistant tuberculosis in South Africa. *PLoS One* 2013; **8**(5): e63057.

17. Dheda K, Shean K, Zumla A, et al. Early treatment outcomes and HIV status of patients with extensively drug-resistant tuberculosis in South Africa: a retrospective cohort study. *Lancet* 2010; **375**(9728): 1798-807.

18. Fox G CV. Unpublished data (Sydney, Australia). 2018.

19. Gegia M, Kalandadze I, Kempker RR, Magee MJ, Blumberg HM. Adjunctive surgery improves treatment outcomes among patients with multidrug-resistant and extensively drug-resistant tuberculosis. *International journal of infectious diseases : IJID : official publication of the International Society for Infectious Diseases* 2012; **16**(5): e391-6.

20. Guglielmetti L, Jaspard M, Le Du D, et al. Long-term outcome and safety of prolonged bedaquiline treatment for multidrug-resistant tuberculosis. *Eur Respir J* 2017; **49**(3).

21. Guglielmetti L, Le Du D, Jachym M, et al. Compassionate use of bedaquiline for the treatment of multidrug-resistant and extensively drug-resistant tuberculosis: interim analysis of a French cohort. *Clin Infect Dis* 2015; **60**(2): 188-94.

22. Guglielmetti L. Unpublished data (Paris, France). 2018.

23. Hughes J, Isaakidis P, Andries A, et al. Linezolid for multidrug-resistant tuberculosis in HIV-positive and -uninfected patients. *Eur Respir J* 2015; **46**(1): 271-4.

24. Isaakidis P, Varghese B, Mansoor H, et al. Adverse events among HIV/MDR-TB co-infected patients receiving antiretroviral and second line anti-TB treatment in Mumbai, India. *PLoS One* 2012; **7**(7): e40781.

25. Jarlsberg L, Nahid P. Unpublished data (San Francisco, USA). 2016.

26. Kempker RR, Kipiani M, Mirtskhulava V, Tukvadze N, Magee MJ, Blumberg HM. Acquired Drug Resistance in Mycobacterium tuberculosis and Poor Outcomes among Patients with Multidrug-Resistant Tuberculosis. *Emerg Infect Dis* 2015; **21**(6): 992-1001.

27. Charles M, Vilbrun SC, Koenig SP, et al. Treatment outcomes for patients with multidrug-resistant tuberculosis in post-earthquake Port-au-Prince, Haiti. *The American journal of tropical medicine and hygiene* 2014; **91**(4): 715-21.

28. Jeong BH, Jeon K, Park HY, et al. Outcomes of pulmonary MDR-TB: impacts of fluoroquinolone resistance and linezolid treatment. *The Journal of antimicrobial chemotherapy* 2015; **70**(11): 3127-33.

29. Koh WJ, Kang YR, Jeon K, et al. Daily 300 mg dose of linezolid for multidrug-resistant and extensively drug-resistant tuberculosis: updated analysis of 51 patients. *The Journal of antimicrobial chemotherapy* 2012; **67**(6): 1503-7.

30. Kuksa L. Unpublished data (Latvia). 2018.

31. Kvasnovsky CL, Cegielski JP, van der Walt ML. Treatment Outcomes for Patients with Extensively Drug-Resistant Tuberculosis, KwaZulu-Natal and Eastern Cape Provinces, South Africa. *Emerg Infect Dis* 2016; **22**(9).

32. Kvasnovsky CL, Cegielski JP, Erasmus R, Siwisa NO, Thomas K, der Walt ML. Extensively drug-resistant TB in Eastern Cape, South Africa: high mortality in HIV-negative and HIV-positive patients. *Journal of acquired immune deficiency syndromes (1999)* 2011; **57**(2): 146-52.

33. Eker B, Ortmann J, Migliori GB, et al. Multidrug- and extensively drug-resistant tuberculosis, Germany. *Emerg Infect Dis* 2008; **14**(11): 1700-6.

34. Laniado-Laborin R, Estrada-Guzman J, Perez H, Batiz-Armenta F, Alcantar-Schramm JM. Treatment of multidrug-resistant tuberculosis in a high-prevalence region through a binational consortium. *Int J Tuberc Lung Dis* 2012; **16**(5): 610-1.

35. Chang KC, Yew WW, Cheung SW, et al. Can intermittent dosing optimize prolonged linezolid treatment of difficult multidrug-resistant tuberculosis? *Antimicrobial agents and chemotherapy* 2013; **57**(7): 3445-9.

36. Chang KC, Leung CC, Yew WW, et al. Pyrazinamide may improve fluoroquinolone-based treatment of multidrug-resistant tuberculosis. *Antimicrobial agents and chemotherapy* 2012; **56**(11): 5465-75.

37. Marks SM, Flood J, Seaworth B, et al. Treatment practices, outcomes, and costs of multidrug-resistant and extensively drug-resistant tuberculosis, United States, 2005-2007. *Emerg Infect Dis* 2014; **20**(5): 812-21.

38. Tiberi S, Payen MC, Sotgiu G, et al. Effectiveness and safety of meropenem/clavulanate-containing regimens in the treatment of MDR- and XDR-TB. *Eur Respir J* 2016; **47**(4): 1235-43.

39. Tiberi S, Sotgiu G, D'Ambrosio L, et al. Comparison of effectiveness and safety of imipenem/clavulanate- versus meropenem/clavulanate-containing regimens in the treatment of MDR- and XDR-TB. *Eur Respir J* 2016; **47**(6): 1758-66.

40. Borisov SE, Dheda K, Enwerem M, et al. Effectiveness and safety of bedaquiline-containing regimens in the treatment of MDR- and XDR-TB: a multicentre study. *Eur Respir J* 2017; **49**(5).

41. Milanov V, Falzon D, Zamfirova M, et al. Factors associated with treatment success and death in cases with multidrug-resistant tuberculosis in Bulgaria, 2009-2010. *International journal of mycobacteriology* 2015; **4**(2): 131-7.

42. Ndjeka N, Conradie F, Schnippel K, et al. Treatment of drug-resistant tuberculosis with bedaquiline in a high HIV prevalence setting: an interim cohort analysis. *Int J Tuberc Lung Dis* 2015; **19**(8): 979-85.

43. Ndjeka N. Unpublished data (South Africa). 2018.

44. O'Donnell MR, Padayatchi N, Kvasnovsky C, Werner L, Master I, Horsburgh CR, Jr. Treatment outcomes for extensively drug-resistant tuberculosis and HIV co-infection. *Emerg Infect Dis* 2013; **19**(3): 416-24.

45. Palmero D, Gonzalez Montaner P, Cufre M, Garcia A, Vescovo M, Poggi S. First series of patients with XDR and pre-XDR TB treated with regimens that included meropenen-clavulanate in Argentina. *Archivos de bronconeumologia* 2015; **51**(10): e49-52.

46. Podewils LJ, Gler MT, Quelapio MI, Chen MP. Patterns of treatment interruption among patients with multidrug-resistant TB (MDR TB) and association with interim and final treatment outcomes. *PLoS One* 2013; **8**(7): e70064.

47. Riekstina V, Leimane V, Cirule A, Kuksa L, Latvia National TB registry. Unpublished data (Latvia). 2016.

48. Rodrigues D. Unpublished data (Brazil). 2018.

49. Seo H. Unpublished data (Seoul, South Korea). 2018.

50. Seung KJ, Franke M, Linton SW. Multidrug-Resistant Tuberculosis Treatment in North Korea: Is Scale-Up Possible? *PLoS medicine* 2016; **13**(8): e1002062.

51. Jo KW, Lee SD, Kim WS, Kim DS, Shim TS. Treatment outcomes and moxifloxacin susceptibility in ofloxacin-resistant multidrug-resistant tuberculosis. *Int J Tuberc Lung Dis* 2014; **18**(1): 39-43.

52. Singla R, Caminero JA, Jaiswal A, et al. Linezolid: an effective, safe and cheap drug for patients failing multidrug-resistant tuberculosis treatment in India. *Eur Respir J* 2012; **39**(4): 956-62.

53. Skrahina A. Unpublished data (Minsk, Belarus). 2018.

54. Smith SE, Ershova J, Vlasova N, et al. Risk factors for acquisition of drug resistance during multidrug-resistant tuberculosis treatment, Arkhangelsk Oblast, Russia, 2005-2010. *Emerg Infect Dis* 2015; **21**(6): 1002-11.

55. Diacon AH, Pym A, Grobusch MP, et al. Multidrug-resistant tuberculosis and culture conversion with bedaquiline. *N Engl J Med* 2014; **371**(8): 723-32.

56. Diacon AH, Pym A, Grobusch M, et al. The diarylquinoline TMC207 for multidrug-resistant tuberculosis. *N Engl J Med* 2009; **360**(23): 2397-405.

57. Pym AS, Diacon AH, Tang SJ, et al. Bedaquiline in the treatment of multidrug- and extensively drug-resistant tuberculosis. *Eur Respir J* 2016; **47**(2): 564-74.

58. Udwadia ZF, Sen T, Moharil G. Assessment of linezolid efficacy and safety in MDR- and XDR-TB: an Indian perspective. *Eur Respir J* 2010; **35**(4): 936-8; author reply 8-40.

59. van Altena R, de Vries G, Haar CH, et al. Highly successful treatment outcome of multidrug-resistant tuberculosis in the Netherlands, 2000-2009. *Int J Tuberc Lung Dis* 2015; **19**(4): 406-12.

60. Vasilyeva I. Unpublished data (Russia). 2018.

61. Viiklepp P, Estonian TB Registry. Unpublished data (Estonia). 2016.

62. Kwak N, Kim HR, Yoo CG, Kim YW, Han SK, Yim JJ. Changes in treatment outcomes of multidrug-resistant tuberculosis. *Int J Tuberc Lung Dis* 2015; **19**(5): 525-30.

63. Achar J. Unpublished data (Uzbekistan). 2019.

64. Isaakidis P. Unpublished data (Mumbai). 2019.

65. Skrahina A. Unpublished data (Belarus). 2019.
