# Supplementary material for: Identifying patients with multidrug-resistant tuberculosis who may benefit from shorter durations of treatment
Source: PLoS One. 2023 Oct 5;18(10):e0292106. doi: 10.1371/journal.pone.0292106 (PMC10553332; doi:10.1371/journal.pone.0292106)
Supplement: Attachment — Submitted filename: PLOS ONE response to reviewers R1.docx [file pone.0292106.s002.docx]

Dear Dr. Gotham,

Thanks for your email of Aug 7, 2023.

We have revised the manuscript as suggested by yourself and the external reviewers, and are re-submitting this manuscript. As requested below we have included a marked-up copy of text with tables embedded, plus a clean copy of same, as well as a point-by-point response to all comments, and a new figure presenting a timeline of changes in WHO recommendations.

Please note that we have included an updated Supplemental file as well.

We hope this is now acceptable for publication in PLOS One and look forward to hearing from you.

N Winters and D Menzies, on behalf of all authors

Dear Dr. Winters,

Thank you for submitting your manuscript to PLOS ONE. After careful consideration, we feel that it has merit but does not fully meet PLOS ONE’s publication criteria as it currently stands. Therefore, we invite you to submit a revised version of the manuscript that addresses the points raised during the review process.

Please see the comments from reviewers, below.

Please submit your revised manuscript by Sep 21 2023 11:59PM. If you will need more time than this to complete your revisions, please reply to this message or contact the journal office at [plosone@plos.org](mailto:plosone@plos.org). When you're ready to submit your revision, log on to https://www.editorialmanager.com/pone/ and select the 'Submissions Needing Revision' folder to locate your manuscript file.

Please include the following items when submitting your revised manuscript:

- A rebuttal letter that responds to each point raised by the academic editor and reviewer(s). You should upload this letter as a separate file labeled 'Response to Reviewers'.
- A marked-up copy of your manuscript that highlights changes made to the original version. You should upload this as a separate file labeled 'Revised Manuscript with Track Changes'.
- An unmarked version of your revised paper without tracked changes. You should upload this as a separate file labeled 'Manuscript'.

If you would like to make changes to your financial disclosure, please include your updated statement in your cover letter. Guidelines for resubmitting your figure files are available below the reviewer comments at the end of this letter.

If applicable, we recommend that you deposit your laboratory protocols in protocols.io to enhance the reproducibility of your results. Protocols.io assigns your protocol its own identifier (DOI) so that it can be cited independently in the future. For instructions see: [https://journals.plos.org/plosone/s/submission-guidelines#loc-laboratory-protocols](https://can01.safelinks.protection.outlook.com/?url=https%3A%2F%2Fjournals.plos.org%2Fplosone%2Fs%2Fsubmission-guidelines%23loc-laboratory-protocols&data=05%7C01%7Cnicholas.winters%40mail.mcgill.ca%7Cccda2568be8847a721ca08db97b17091%7Ccd31967152e74a68afa9fcf8f89f09ea%7C0%7C0%7C638270560732606623%7CUnknown%7CTWFpbGZsb3d8eyJWIjoiMC4wLjAwMDAiLCJQIjoiV2luMzIiLCJBTiI6Ik1haWwiLCJXVCI6Mn0%3D%7C3000%7C%7C%7C&sdata=OkSkkECdG4hRZYT8%2FwkZ%2Fry0xyPK7Egl4uvd38Sy5Nw%3D&reserved=0). Additionally, PLOS ONE offers an option for publishing peer-reviewed Lab Protocol articles, which describe protocols hosted on protocols.io. Read more information on sharing protocols at [https://plos.org/protocols?utm_medium=editorial-email&utm_source=authorletters&utm_campaign=protocols](https://can01.safelinks.protection.outlook.com/?url=https%3A%2F%2Fplos.org%2Fprotocols%3Futm_medium%3Deditorial-email%26utm_source%3Dauthorletters%26utm_campaign%3Dprotocols&data=05%7C01%7Cnicholas.winters%40mail.mcgill.ca%7Cccda2568be8847a721ca08db97b17091%7Ccd31967152e74a68afa9fcf8f89f09ea%7C0%7C0%7C638270560732606623%7CUnknown%7CTWFpbGZsb3d8eyJWIjoiMC4wLjAwMDAiLCJQIjoiV2luMzIiLCJBTiI6Ik1haWwiLCJXVCI6Mn0%3D%7C3000%7C%7C%7C&sdata=v9rxGH6VOfx2B%2BU3xDmdK%2FA8Nc9IWHtahImqIQ7NpE4%3D&reserved=0).

We look forward to receiving your revised manuscript.

Kind regards,

Dzintars Gotham
Academic Editor
PLOS ONE

Journal requirements:

When submitting your revision, we need you to address these additional requirements.

1. Please ensure that your manuscript meets PLOS ONE's style requirements, including those for file naming. The PLOS ONE style templates can be found at

https://journals.plos.org/plosone/s/file?id=wjVg/PLOSOne_formatting_sample_main_body.pdf and

<https://journals.plos.org/plosone/s/file?id=ba62/PLOSOne_formatting_sample_title_authors_affiliations.pdf>

**Response: the paper has been formatted to meet the above outlined requirements, tables appear after they are cited within the text.**

2. Please provide additional details regarding participant consent. In the ethics statement in the Methods and online submission information, please ensure that you have specified (1) whether consent was informed and (2) what type you obtained (for instance, written or verbal, and if verbal, how it was documented and witnessed). If your study included minors, state whether you obtained consent from parents or guardians. If the need for consent was waived by the ethics committee, please include this information.

If you are reporting a retrospective study of medical records or archived samples, please ensure that you have discussed whether all data were fully anonymized before you accessed them and/or whether the IRB or ethics committee waived the requirement for informed consent. If patients provided informed written consent to have data from their medical records used in research, please include this information.

**Response: Our ethics statement and participant consent has been included in the methods.**

3. We note that you have indicated that data from this study are available upon request. PLOS only allows data to be available upon request if there are legal or ethical restrictions on sharing data publicly. For more information on unacceptable data access restrictions, please see http://journals.plos.org/plosone/s/data-availability#loc-unacceptable-data-access-restrictions.

In your revised cover letter, please address the following prompts:

a) If there are ethical or legal restrictions on sharing a de-identified data set, please explain them in detail (e.g., data contain potentially sensitive information, data are owned by a third-party organization, etc.) and who has imposed them (e.g., an ethics committee). Please also provide contact information for a data access committee, ethics committee, or other institutional body to which data requests may be sent.

b) If there are no restrictions, please upload the minimal anonymized data set necessary to replicate your study findings as either Supporting Information files or to a stable, public repository and provide us with the relevant URLs, DOIs, or accession numbers. For a list of acceptable repositories, please see http://journals.plos.org/plosone/s/data-availability#loc-recommended-repositories.

We will update your Data Availability statement on your behalf to reflect the information you provide.

**Response: The data is accessible through University College London who now hold the data as a publicly accessible repository. Access to this is governed by an oversight committee, and applications must be made to that committee. There are legal restrictions in terms of signed data sharing agreements with all data contributors. So, application must be made to access the data. But it is publicly accessible and inquiries can be made through this link:** [**https://www.ucl.ac.uk/global-health/research/tb-ipd-platform**](https://www.ucl.ac.uk/global-health/research/tb-ipd-platform)

4. Please include your full ethics statement in the ‘Methods’ section of your manuscript file. In your statement, please include the full name of the IRB or ethics committee who approved or waived your study, as well as whether or not you obtained informed written or verbal consent. If consent was waived for your study, please include this information in your statement as well.

**Response: Our ethics statement and participant consent has been included in the methods.**

5. Please include captions for your Supporting Information files at the end of your manuscript, and update any in-text citations to match accordingly. Please see our Supporting Information guidelines for more information: http://journals.plos.org/plosone/s/supporting-information.

**Response: We have included a list of captions for our supporting material at the end of the manuscript.**

[Note: HTML markup is below. Please do not edit.]

Reviewers' comments:

Reviewer's Responses to Questions

**Comments to the Author**

1. Is the manuscript technically sound, and do the data support the conclusions?

The manuscript must describe a technically sound piece of scientific research with data that supports the conclusions. Experiments must have been conducted rigorously, with appropriate controls, replication, and sample sizes. The conclusions must be drawn appropriately based on the data presented.

Reviewer #1: Partly

Reviewer #2: Yes

Reviewer #3: Yes

2. Has the statistical analysis been performed appropriately and rigorously?

Reviewer #1: Yes

Reviewer #2: Yes

Reviewer #3: Yes

3. Have the authors made all data underlying the findings in their manuscript fully available?

The [PLOS Data policy](https://can01.safelinks.protection.outlook.com/?url=http%3A%2F%2Fwww.plosone.org%2Fstatic%2Fpolicies.action%23sharing&data=05%7C01%7Cnicholas.winters%40mail.mcgill.ca%7Cccda2568be8847a721ca08db97b17091%7Ccd31967152e74a68afa9fcf8f89f09ea%7C0%7C0%7C638270560732606623%7CUnknown%7CTWFpbGZsb3d8eyJWIjoiMC4wLjAwMDAiLCJQIjoiV2luMzIiLCJBTiI6Ik1haWwiLCJXVCI6Mn0%3D%7C3000%7C%7C%7C&sdata=Lc2eUb%2FVkcCpTezk2rLF8%2BhBLJucfv8pRGHTxQBO9Sg%3D&reserved=0) requires authors to make all data underlying the findings described in their manuscript fully available without restriction, with rare exception (please refer to the Data Availability Statement in the manuscript PDF file). The data should be provided as part of the manuscript or its supporting information, or deposited to a public repository. For example, in addition to summary statistics, the data points behind means, medians and variance measures should be available. If there are restrictions on publicly sharing data—e.g. participant privacy or use of data from a third party—those must be specified.

Reviewer #1: Yes

Reviewer #2: Yes

Reviewer #3: Yes

4. Is the manuscript presented in an intelligible fashion and written in standard English?

PLOS ONE does not copyedit accepted manuscripts, so the language in submitted articles must be clear, correct, and unambiguous. Any typographical or grammatical errors should be corrected at revision, so please note any specific errors here.

Reviewer #1: Yes

Reviewer #2: Yes

Reviewer #3: Yes

5. Review Comments to the Author

Please use the space provided to explain your answers to the questions above. You may also include additional comments for the author, including concerns about dual publication, research ethics, or publication ethics. (Please upload your review as an attachment if it exceeds 20,000 characters)

**Reviewer #1**: There has been considerable work done by the research team working with large data sets with the methods well articulated. The data sets included cover a large date range for the studies (between 1993 and 2019) when there has been considerable changes in WHO recommended treatments and diagnosis for MDRTB, including the use of rapid molecular diagnostic tests for diagnosis of Rif Resistant TB (Xpert), the introduction of Bedaquilline in 2013 and the 9 month regimen in 2016. The article would benefit in highlighting the timelines of key changes in WHO guidance with regards to MDRTB treatment to help orientate the reader - potential a figure/timeline of guidance with regards to priority/group A drugs, recommended durations and introduction of new drugs. If the majority of the earlier studies were excluded in the process outlined in Fig 1 that would be interesting information to know given the progression of the field since 1993.

**Response: We thank the reviewer for their comment, and we agree that a figure outlining key changes would be helpful, which we have added to the supplement (also see below)**

**See lines 126 to 127: *“(Supplemental Figure S1 presents a timeline for important changes in WHO treatment guidelines for MDR-TB).”***

**Supplemental Figure S1. Changes in World Health Organization treatment guidelines for multidrug-resistant tuberculosis between 1997 and 2022.**

Abbreviations: mo: months; XDR-TB: extensively drug resistant tuberculosis; Bdq: bedaquiline; Lzd: linezolid; Am: amikacin; Km: kanamycin; BPaL: bedaquiline, pretomanid, and linezolid; BPaLM: bedaquiline, pretomanid, linezolid and moxifloxacin.

Did the researchers see any impact of the guidance of a 9 month regimen in 2016 on the mean duration of treatment between 2016 - 2019 as that was the first time that WHO recommended a duration of treatment less than 18months?

**Response: Regarding mean duration of treatment over time, we did not assess temporal changes as we were attempting to identify patient characteristics for hypothesis generating purposes. Unfortunately, there were few patients included in the study sample (n=223) who were treated in 2016 and later, and it would be difficult to interpret results from such a small sample size when assessing temporal trends.**

For the definition used for extensive disease (line 164) the criteria for CXR matches the WHO definition (Presence of bilateral cavitary disease or extensive parenchymal damage on CXR) but the use of the broad statement of AFB positivity encompasses everything from scanty to 3+ which have different impacts on severity of disease. Did the data sets give the level of smear AFB positivity? If this was given, was scanty/+1 AFB have different durations to 3+ smears? If this is not known, the research team may want to reflect on if this may have had any effect.

**Response: The reviewer brings up an important point, unfortunately we do not have the level of smear positivity in our data and only information as to whether the AFB smear was positive or negative. We have added a sentence in the limitations to acknowledge this.**

**See the limitation section in the discussion, line 392-394: *“******We also did not have data on the number of cavities, only the presence or absence, nor did we have data on level of AFB smear positivity (only positive or negative), and were unable to assess what effect this had on duration.”***

Line 210 -214 - "Use of capreomycin, kanamycin, moxifloxacin, levofloxacin, PAS, linezolid, clofazimine, Amx-Clv, clarithromycin, or bedaquiline, as well as greater number of drugs, were all associated with longer treatment duration." It is not clear what this sentence is saying - if any of these drugs were individually involved in the regimen then the duration was longer? Most of these drugs are key components of DRTB regimens. This statement also seems to conflict with line 217 "Use of bedaquiline was associated with shorter treatment duration by -0.51 (95% CI -0.87 to -0.15) months".

**Response: Thank you for the comment, we have clarified the sentence in the results described below. These results are for the univariable associations, while in the fully adjusted multivariable model bedaquiline was associated with a decrease in treatment duration.**

**Please see line 264-267: *“Within the treatment regimen of a patient, the use of capreomycin, kanamycin, moxifloxacin, levofloxacin, PAS, linezolid, clofazimine, Amx-Clv, clarithromycin, or bedaquiline, as well as greater number of drugs, were all associated with longer treatment duration in univariable analyses.”***

**We also modified the text regarding the reversal of effect for the bedaquiline duration estimates in the adjusted analyses.**

**See lines 287 to 289: *“In contrast to univariable regression results, use of bedaquiline was associated with shorter treatment duration by -0.51 (95% CI -0.87 to -0.15) months in adjusted analyses.”***

For the statement on line 219 "... and with greater number of drugs used, or use of moxifloxacin, kanamycin, capreomycin, or Amx-Clv." given that prior to the introduction of Bdq in 2013, FLQ and SLI (moxi, Kanamycin and capreomycin) were the backbone of any DRTB regimen so is this saying that the use of these more effective drugs were associated with longer durations?

**Response: Thank you for the comment. The result indicates these drugs were associated with longer treatment, when controlling for all other variables in the model. The associations with these drugs should not be interpreted as poorer efficacy, as all patients in the population had successful treatment. Rather, they were just associated with longer treatment duration and as the purpose of this study was hypothesis generation, the reasons for why there was an association with longer treatment should be investigated further.**

In the discussion section the researchers do not mention the latest WHO DRTB guidance (Dec 2022) on the 6 month regimens - BPaLM/BPaL. What does this recommendation for a standard 6 month regimen for BPaLM for adults >14yrs with MDR/RR-TB or with MDR/RR-TB and resistance to fluoroquinolones (pre-XDR-TB), regardless of HIV status mean for the approaches outlined in the article? The BPaLM recommendation does restrict the use of this regimen in those with prior exposure to drugs in the regimen (>1month) so the points made regarding these groups may still be valid but it would be good to see the explicit mention of this dramatic advance in shorter effective treatment for DRTB.

**Response: The reviewer brings up an important point regarding the newest regimens that included pretomanid. As our study was not intended to provided evidence to inform clinical recommendations, nor for individual clinical decisions and our results should not be interpreted in that sense. However, for new studies investigating the use of BPaL/BPaLM, our results may be applicable to those studies, in terms of patient populations that should NOT be excluded. We have made note of this in our discussion:**

**See lines 362-367: *“******Use of bedaquiline was associated with shorter treatment duration across the majority of subgroups, suggesting that inclusion of patients previously excluded from RCTs^9-11,29^ or considered ineligible for short MDR treatment in guidelines^3^ could be included in future trials of shorter bedaquiline and/or pretomanid containing regimens (such as bedaquiline, pretomanid, & linezolid (BPaL), or BPaL plus moxifloxacin, BPaLM) most recently recommended by the WHO^3^.”***

**Reviewer #2:** The authors have taken a novel approach to try to identify factors associated with treatment duration for MDR/RR-TB. This is a hypothesis generating study. The study is well-written, clearly described with appropriate analyses.

Major comments:
As the data involved in the study are from before 2020, and there is no mention in the article of the 6 month all oral regimens, the article doesn’t really address how the approach might be useful in the future for assessing duration – especially if treatment is given as fixed durations based on regimen.

**Response: We appreciate the reviewers point regarding the newest regimens that include pretomanid, which echoes the last point made by Reviewer 1. See our response above. And the revised text lines 362-367 also given above.**

In the supplement 1 under search criteria it states “Studies exclusively in children or of patients treated with short regimens were excluded as these were the topics of two concurrent individual patient data meta-analyses at time of original publication”. If these are indeed excluded it would be helpful to have this stated in the methods. Also then including within the discussion the potential impact or not on the results of this for this methodology.

**Response: We thank the reviewer for their response, we have added mention of this in the methods to highlight the exclusion:**

**See lines 128-129: *“Studies exclusively in children were excluded.”***

**Regarding discussion of populations of children, we have added note of this in our limitations:**

**See lines 394-396 *“Further, studies conducted exclusively in children were excluded from the IPD, and we were not able to assess associations in this population”***

Minor comments:
Introduction:
the guidelines for treatment referenced are older guidelines. Suggest reference up to date guidelines from 2022. Line 74-75 states “Current recommended treatment for advanced and extensive MDR-TB is as long as 18-20 months”. This is no longer the case with the 2022 current guidelines. The recommendation that “patients without extensive TB disease and without severe extrapulmonary TB” should not have shorter regimen applies to the 9-month regimens from the 2019 guideline. Suggest rephrase this sentence to clarify how things apply with the current guidelines.

**Response: We appreciate the comment. We have updated the reference to the latest WHO guidelines (2022) and updated the wording of the recommendation to reflect the eligibility in the 2022 guideline which recommends the 9-month all oral regimen to “those with no extensive or severe TB disease and no severe extrapulmonary TB” as stated in the guideline.**

**See line 88-90: *“Current recommended treatment from the World Health Organization (WHO) for extensive or severe MDR-TB is as long as 18-20 months^3^ and entails a high patient burden”***

Methods
Line 146 spell out abbreviations at first use: 95% confidence interval (95% CI), and line 163 fluoroquinolone (FQ) and second line injectables (SLI), and 288 ART

**Response: Thank you for pointing this out, we have updated the text to include the terms we abbreviated for CI, FQ, SLI, and ART.**

Suggest state more clearly whether when referring to MDR-TB you are being inclusive (MDR/RR-TB) or only including those with both isoniazid and rifampicin resistance. From line 95-96 it suggests that also includes rifampicin resistance, however in Methods only ever state MDR-TB which is defined as resistance to both rifampicin and isoniazid. In line 100 it refers to study dataset described in reference 14 – would be easier if state clearly in methods that actually inclusion criteria was rifampicin resistant TB (RR-TB). Would suggest either changing to using RR-TB throughout or MDR/RR-TB throughout and only use MDR-TB when specifically meaning resistance as per definition in in lines 71-72.

**Response: We appreciate the response and indeed we did include MDR/RR-TB patients. We have updated the methods and results of the text so we refer to MDR/RR-TB where appropriate throughout.**

For the treatment outcomes, 2 references (21 and 22) are mentioned for definitions. These references do have some differences. It would be helpful to the reader to state clearly here whether outcomes given by each site were taken as the outcomes or whether a single set of definitions was retrospectively applied to all date. Also reference 22 is from 2022, while the IPD data was only up til 2019, so not sure if the definitions referred to here were used at all.

**Response: We appreciate the comment. We have updated the reference to reflect the WHO definition (WHO 2013) used in each included study that did not use the Laserson et al. definition. In assembling the IPD datasets we verified outcome definitions used by each author/group and harmonized all to the definitions suggested by WHO in 2013, as described in detail in earlier publications (see detailed tables about this in the supplement of Ahmad et al, Lancet). Additionally, we have added clarification on outcome definitions.**

**See lines 139-143: *“From the included studies, we included only patients that had successful (cured or completed) treatment outcomes, as defined elsewhere^22,23^ and who had their individual treatment duration recorded. We verified outcomes provided by study investigators in their original study, and harmonized these to WHO 2013 definitions,^23^ as detailed elsewhere (see supplement to Ahmad et al.^15^).”***

**Reviewer #3**: Winters et al present an analysis of ecological and individual factors associated with successful shorter treatment of patients with MDR TB. Leveraging data from a previously published IPD meta analysis, the authors evaluated data of 6702 patients from 34 different countries. In a site level, the proportion of resistance to FQ-SILI was associated with longer duration, while in at an individual level an adjusted model identified AFB smear, presence of cavities, HIV status and added resistance as factors associated with longer treatment duration. Importantly, the use of bedaquiline was associated with shorter treatment duration. The study is well written and the statistics are appropriate, the topic is important, some comments below:

1. My main concern is that the study relies on data obtained between 2009 and 2018, at that time longer 15-24 month treatment regimens were recommended by WHO. As the authors are aware, current WHO recommendations endorse the use of shorter 6 month all oral regimens using BPaL/BPaLM thus the data presented here albeit interesting may not be applicable to the current MDR TB scenario. The authors should make a point in the discussion into what settings would your data be applicable? Maybe in patients who fail BPaL/BPaLM? In countries without access to pretomanid or bedaquiline? Or in populations were BPaL is not validated yet such as children, pregnancy, end stage renal disease among others.

**Response: The reviewer raises an important point regarding the newest regimens that include pretomanid, which echoes the same questions as Reviewer 1 (last point) and Reviewer 2 (first point). See our response to Reviewer 1 (last point) above.**

2. Line 77-79 “In the past 10 years, several studies have investigated shorter regimens for treatment “ of MDR-TB in randomized controlled trials (RCTs), but these may not reflect treatment in programmatic settings” This statement is not fully accurate, there is recent data on the applicability of shorter regimens under programmatic conditions (Haley et al pmid: 37249079, Acuna-Villaorduna et al pmid: 37491751). In the US, outcomes of all oral shorter regimens report > 95% treatment success.

**Response: We appreciate the reviewer’s comment. Here we were referring to the follow-up and intensity of care that are common in RCTs, compared to programmatic settings. However, we have added mention of the potential success of these regimens when used in programmatic settings and reference to the studies you have mentioned in our discussion.**

**See lines 427-431: *“Although RCTs can provide clearer evidence on optimal duration, these are expensive, time consuming, often lack generalizability, and can test only a limited number of durations and/or regimens at once. As well, evidence from cohorts are useful as potential indicators of success of these regimens in programmatic settings^32,33^).”***

3. The authors included patients with successful treatment outcomes in order to avoid bias which is a reasonable approach, however it could have introduced selection bias. Some of the treatment failure patients who were excluded could have been due to medication side effects and in fact these group may benefit even more of shorter regimens. In fact, in the TB PRACTECAL study, side effects were the main cause (49%) of treatment discontinuation. The authors could consider adding this as a potential limitation

**Response: We thank the reviewer for their comment. Indeed, this was a concern of ours in selecting our population of only those who had successful treatment. In our methods we outlined that we assessed the impact this may have on our estimates by analyzing the same model but with inverse probability of selection weights that used a propensity score predicting selection into our study population. We observed no substantial differences between weighted and unweighted estimates (see Results lines 298-301 and Supplemental Table S3).**

**However, the effect that adverse events may have on negative treatment outcomes may still have an impact on results, as such we have included mention of this in our discussion.**

**See lines 403-418:** ***“Because our population included only those with treatment success, our findings may not be generalizable to all patients with MDR/RR-TB, although we conducted inverse probability of selection weighted analyses which indicated no substantial differences in the excluded vs included populations. However, adverse events may be more common with longer treatment, and contribute to treatment failure (and as such were excluded in this study) yet it is possible that patients with adverse events would benefit even more from shorter treatment.”***

4. The authors could expand more on the outcome definition: successful treatment. They refer to the WHO 2022 document (ref 22) who is more an update on shorter regimens and briefly discuss treatment outcomes definition but specifically for all oral shorter regimens which are not the ones the authors evaluate in their analysis. I suggest the authors to add in the text the precise definition of successful outcome they used (cure (negative cultures) without relapse if evaluated at a given time for instance) and how they managed the differences in outcome definitions between different studies in the IPD datasets.

**Response: As the reviewer points out, definitions of MDR-TB treatment outcomes have changed over time, particularly with the new short regimens. In assembling the IPD datasets we verified outcome definitions used by each author/group and harmonized all to the definitions suggested by WHO in 2013, as described in detail in earlier publications (see detailed tables about this in the supplement of Ahmad et al, Lancet).**

**See lines 139-143: *“From the included studies, we included only patients that had successful (cured or completed) treatment outcomes, as defined elsewhere^22,23^ and who had their individual treatment duration recorded. We verified outcomes provided by study investigators in their original study, and harmonized these to WHO 2013 definitions,^23^ as detailed elsewhere (see supplement to Ahmad et al.^15^).”***

5. The data on bedaquiline is interesting, I would emphasize more on this. It is not surprising to me that bedaquiline is associated with shorter duration, bedaquiline is highly bactericidal against M. tuberculosis (Yamada et al pmid 36165631) and in animal models can eradicate MTB in 3-4 weeks when used in combination with pretomanid and moxifloxacin. I think the authors could emphasize that their data support the use of bedaquiline for all MDR TB patients.

**Response: We thank the reviewer for their comment, and we agree. Bedaquiline is essential in treatment of all MDR-TB patients. We have added emphasis of this in our discussion, but as our study is hypothesis generating we have stated this:**

**See lines 355-357: *“******Our finding that treatment duration is shorter when bedaquiline was used, is supported by several studies that have established the efficacy of bedaquiline,^3,15,28^ which further supports the use of bedaquiline containing regimens for all MDR/RR-TB patients.”***

6. SILI abbreviation (line 164) could be defined, I believe it is second line injectables

**Response: Thank you for pointing this out, we have added the definition along with the abbreviation for SLI.**

6. PLOS authors have the option to publish the peer review history of their article ([what does this mean?](https://can01.safelinks.protection.outlook.com/?url=https%3A%2F%2Fjournals.plos.org%2Fplosone%2Fs%2Feditorial-and-peer-review-process%23loc-peer-review-history&data=05%7C01%7Cnicholas.winters%40mail.mcgill.ca%7Cccda2568be8847a721ca08db97b17091%7Ccd31967152e74a68afa9fcf8f89f09ea%7C0%7C0%7C638270560732606623%7CUnknown%7CTWFpbGZsb3d8eyJWIjoiMC4wLjAwMDAiLCJQIjoiV2luMzIiLCJBTiI6Ik1haWwiLCJXVCI6Mn0%3D%7C3000%7C%7C%7C&sdata=qG3gMJeWnkF9talizCT4SThldUiQmTz5pXwTBVjug%2B4%3D&reserved=0)). If published, this will include your full peer review and any attached files.


If you choose “no”, your identity will remain anonymous but your review may still be made public.


**Do you want your identity to be public for this peer review?** For information about this choice, including consent withdrawal, please see our [Privacy Policy](https://can01.safelinks.protection.outlook.com/?url=https%3A%2F%2Fwww.plos.org%2Fprivacy-policy&data=05%7C01%7Cnicholas.winters%40mail.mcgill.ca%7Cccda2568be8847a721ca08db97b17091%7Ccd31967152e74a68afa9fcf8f89f09ea%7C0%7C0%7C638270560732606623%7CUnknown%7CTWFpbGZsb3d8eyJWIjoiMC4wLjAwMDAiLCJQIjoiV2luMzIiLCJBTiI6Ik1haWwiLCJXVCI6Mn0%3D%7C3000%7C%7C%7C&sdata=OF4fO5hJLGqaRFHPMA%2F41PfC8tDPzAbSsQELbMTBTzQ%3D&reserved=0).

Reviewer #1: No

Reviewer #2: No

Reviewer #3: **Yes:** Carlos Acuna-Villaorduna
